# Melanocortin-3 receptors expressed in Nkx2.1(+ve) neurons are sufficient for controlling appetitive responses to hypocaloric conditioning

**DOI:** 10.1038/srep44444

**Published:** 2017-03-15

**Authors:** Clémence Girardet, Maria M. Mavrikaki, Joseph R. Stevens, Courtney A. Miller, Daniel L. Marks, Andrew A. Butler

**Affiliations:** 1 Department of Pharmacology & Physiology, Saint Louis University, Saint Louis, MO 63104, USA; 2 Departments of Metabolism and Aging and Neuroscience, The Scripps Research Institute, Jupiter, FL 33458, USA; 3 Papé Family Pediatric Research Institute, Oregon Health & Science University, Portland, OR 97239, USA

## Abstract

Melanocortin-3 receptors (MC3R) have a contextual role in appetite control that is amplified with hypocaloric conditioning. C57BL/6J (B6) mice subjected to hypocaloric feeding schedules (HFS) exhibit compulsive behavioral responses involving food anticipatory activity (FAA) and caloric loading following food access. These homeostatic responses to calorie-poor environs are attenuated in B6 mice in which Mc3r transcription is suppressed by a lox-stop-lox sequence in the 5’UTR (*Mc3r^TB/TB^*). Here, we report that optimization of caloric loading in B6 mice subject to HFS, characterized by increased meal size and duration, is not observed in *Mc3r^TB/TB^* mice. Analysis of hypothalamic and neuroendocrine responses to HFS throughout the light-dark cycle suggests uncoupling of hypothalamic responses involving appetite-stimulating fasting-responsive hypothalamic neurons expressing agouti-related peptide (AgRP) and neuropeptide Y (Npy). Rescuing Mc3rs expression in Nkx2.1(+ve) neurons is sufficient to restore normal hypothalamic responses to negative energy balance. In addition, *Mc3rs* expressed in Nkx2.1(+ve) neurons are also sufficient to restore FAA and caloric loading of B6 mice subjected to HFS. In summary, MC3Rs expressed in Nkx2.1(+ve) neurons are sufficient to coordinate hypothalamic response and expression of compulsive behavioral responses involving meal anticipation and consumption of large meals during situations of prolonged negative energy balance.

Neural nutrient-sensing networks projecting from control nodes in the hypothalamus and brainstem increase chances of surviving nutrient scarcity by increasing feeding-related motivation while conserving energy^1^,^2^. Studying these systems is clinically relevant, as the appetitive responses controlled by these neurons limit the efficacy of obesity treatments using self-governed hypocaloric diets and bariatric surgery^3^,^4^. Central nervous melanocortin system neurons in the hypothalamus and brainstem are focal points for internal cues of metabolic condition^5^. Much attention has been given to how neural melanocortin-4 receptors (MC4R) optimize appetite and energy expenditure to conserve body mass in hypo- and hyper-caloric dietary conditions^6^. However, melanocortin-3 receptors (MC3R) expressed in hypothalamic and limbic structures also control appetitive behaviors^7^,^8^,^9^,^10^,^11^,^12^,^13^,^14^,^15^,^16^,^17^,^18^. Food anticipatory activity (FAA), a progressive increase in movement anticipating nutrient availability, is attenuated in *Mc3r-*deficient mice^7^,^8^,^10^,^11^. FAA indicates optimization of foraging behavior during times of nutrient scarcity, and involves synchronizing peak rhythms in activity with nutrient availability using circadian (“time-keeping”) functions^19^. MC3Rs expressed in limbic structures regulate feeding-related motivational responses during weight loss^14^, and exert sex-specific control over reward-related behaviors^13^. Hypothalamic and adrenal responses to fasting are also attenuated in *Mc3r*-deficient mice^12^. While MC4R signaling also regulates appetite^6^, it cannot compensate for loss of MC3Rs in hypocaloric situations.

While the mechanisms underlying the behavioral phenotype of *Mc3r-*deficient mice are unclear, abnormal responses of ‘first-order’ melanocortin neurons to sensory inputs may be a critical factor^10^,^12^. Fasting normally activates GABAergic neurons residing in the arcuate nucleus of the hypothalamus (ARC) co-expressing the orexigenic neuropeptide agouti-related peptide (AgRP) and neuropeptide y (Npy). AgRP is an MC3R/MC4R inverse agonist/antagonist; both AgRP and NPY are potent orexigens. Ablating AgRP/Npy neurons in adult mice causes anorexia^20^,^21^,^22^, and compromises adaptation to hypocaloric conditioning^23^. On the other hand, chemo- or opto-genetic activation rapidly induces feeding in mice^24^,^25^. Increased *AgRP* and *Npy* expression in response to fasting^12^ and hypocaloric conditioning^10^ is markedly attenuated in *Mc3r-*deficient mice. While it has not been determined whether this correlates with reduced neural activity, this result could nevertheless imply a complete or partial desensitization to internal cues of metabolic condition. Desensitization of AgRP/Npy neurons in *Mc3r*-deficient mice would also explain lack of compensation by MC4Rs, and other orexigenic neuropeptides/neurotransmitters released by AgRP/Npy neurons^26^, in hypocaloric situations.

Here we further investigated the role of neural MC3Rs in the expression of adaptive responses to hypocaloric conditions. Analysis of gross food intake suggests compulsive behavioral responses involving consumption of large meals in hypocaloric situations requires MC3Rs^9^. We therefore compared meal structure in *Mc3r-*deficient mice and controls in situations of *ad libitum* and hypocaloric feeding conditions. *Mc3r* expression is concentrated in hypothalamic and limbic structures^13^,^14^,^27^. Rescuing *Mc3r* expression in mesolimbic dopamine neurons only partially rescues the expression of homeostatic responses to hypocaloric conditions, improving motivation to self-administer food rewards without affecting gorging^14^. To decipher the contribution of MC3Rs expressed within hypothalamic nuclei to these responses, we rescued *Mc3r* transcription in LoxTB*Mc3r* mice^8^ using transgenic mice expressing Cre in Nkx2.1(+ve) neurons^28^,^29^. Nkx2.1 (also known as thyroid transcription factor 1) is a transcription factor controlling development of the ventral hypothalamus and telencephalon in the CNS, and the thyroid, pituitary and lung in the periphery^30^,^31^. The Nkx2.1-Cre line has previously been used by others to investigate nutrient-responsive signaling pathways in hypothalamic and preoptic areas^32^,^33^,^34^. Our results suggest that MC3Rs expressed in Nkx2.1(+ve) neurons are sufficient for coordinating appetitive and hypothalamic responses to internal cues of metabolic state, but are insufficient for normal nutrient partitioning.

## Results

### Optimizing caloric loading performance during HFS requires MC3Rs

**Figure 1:**
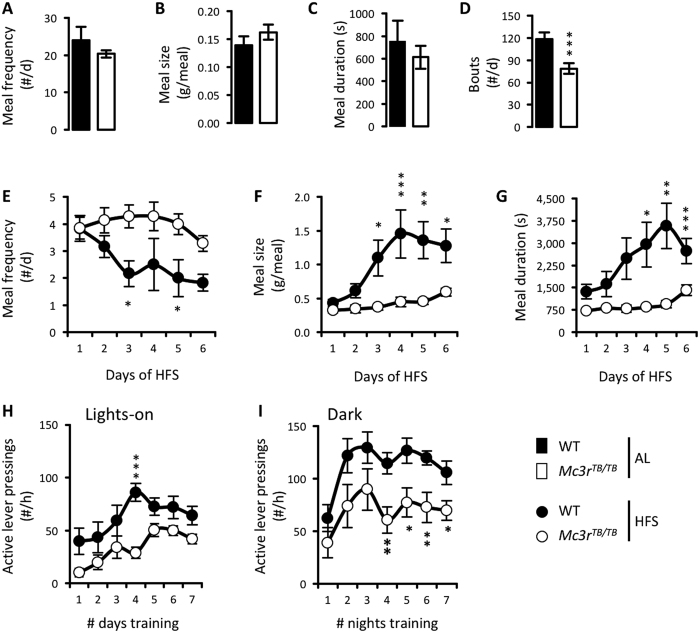
Analysis of meal structure and food self-administrations in male WT and *Mc3r*^*TB/TB*^ mice. Baseline data are averaged over 2 d after 10d acclimation **(A–D),** or during HFS (2.2 g/d presented at ZT4) **(E–G)**. **(A)** Meal frequency, **(B)** meal size, **(C)** meal duration and **(D)** number of bouts data were averaged from *ad libitum* feeding data recorded on day 11 and 12, presented as mean ± SEM. In (**A–D)**, black bars = WT mice, n = 6; white bars = *Mc3r*^*TB/TB*^ mice, n = 7. **(E–G)**. After acclimation and measurement of baseline data, food was removed and mice subject to HFS. **(E)** Meal frequency, **(F)** meal size and **(G)** meal duration are shown for each day during the HFS. For (**E–G**), Two-way ANOVA with repeated measures indicated significant genotype effects on meal number, size and duration (all p < 0.05); a significant effect of time (# days on HFS) on meal frequency (p < 0.01), size and duration (both p < 0.001); and an interaction between genotype and time for meal number (p < 0.05), size and duration (both p < 0.001). **(H,I)** Food self-administration by lever pressing in WT and *Mc3r*^*TB/TB*^ mice trained during the lights-on period (**H**) or dark period (**I**). Food self-administration is shown for mice subjected to HFS. Two-way ANOVA with repeated measures revealed a significant genotype effect (lights-on period, p < 0. 001; dark period, p < 0. 05) and training condition (lights-on or dark) effect on active lever pressings (lights-on period, p < 0. 001; dark period, p < 0. 001), with no interaction between genotype and training condition. *p < 0.05. **p < 0.01. ***p < 0.001. Black circles = WT mice; white circles = *Mc3r*^*TB/TB*^ mice, n = 8/group.

Previous studies examining feeding behavior in *Mc3r-*deficient mice reported gross intake, but did not assess meal structure^9^,^12^,^14^,^15^,^35^,^36^,^37^,^38^. We therefore first compared the effect of *Mc3r*-deficiency on meal structure, using male *Mc3r*^*TB/TB*^ mice and WT controls. At baseline, *Mc3r*^*TB/TB*^ mice weighed more than controls (30.2 ± 0.9 vs. 25.7 ± 0.7 g, p < 0.01) due to increased fat mass (FM) and a tendency for reduced fat-free mass (FFM) (FM 8.5 ± 0.5 vs. 6.0 ± 0.6 g, p < 0.05; FFM 14.8 ± 0.5 vs. 16.6 ± 0.5 g, values are estimated marginal means derived from ANCOVA using total body mass as a covariate). Baseline food intake in the *ad libitum* condition was similar when expressed in g/d (3.1 ± 0.3 vs. 3.0 ± 0.3 g), or adjusted for body mass and/or body composition (data not shown). Meal frequency, size and duration were not affected by genotype in the *ad libitum* condition (Fig. 1A–C), although bout frequency was significantly lower in *Mc3r*^*TB/TB*^ mice (Fig. 1D, p < 0.001). While WT and *Mc3r*^*TB/TB*^ mice ate the same total amount of food each day during HFS (data not shown), their feeding patterns were markedly different (Fig. 1E–G). WT mice responded to HFS by gorging, reducing meal frequency (Fig. 1E) and increasing meal size (Fig. 1F) and duration (Fig. 1G); these responses were all markedly attenuated in *Mc3r*^*TB/TB*^ mice (Fig. 1E–G).

We also assessed food self-administration by lever pressing in WT and *Mc3r*^*TB/TB*^ mice maintained in situations of negative energy balance. As previously reported^14^, *Mc3r*^*TB/TB*^ mice exhibited reduced self-administration of food rewards during training sessions in the lights-on period (Fig. 1H). To control for possible circadian-related phenotype effects^7^,^11^, a separate group of mice trained during the dark period was examined; the phenotype was not affected by lighting schedule (Fig. 1I). Reduced appetite observed in *Mc3r-*deficient mice thus appears to be independent of the time of day at which food is available.

### Neuroendocrine responses to HFS are preserved in Mc3r-deficient mice

We next assessed whether *Mc3r* genotype alters circadian and non-circadian responses to HFS. The response of neuroendocrine hormones to phase shifts in caloric intake exhibits circadian and non-circadian (meal-related) patterns^19^. Changes in hormonal levels preceding meal time may signal impending caloric load, priming the digestive system to minimize disruption of nutrient homeostasis. We bred large numbers of age and sex-matched *Mc3r*^*TB/TB*^ and littermate controls to allow for a comparison daily rhythms in major neuroendocrine hormones that control ingestive behavior and metabolism, and to compare hypothalamic gene expression as a measure of the neuroendocrine responses to altered metabolic condition associated with HFS.

At baseline, there was a modest but highly significant effect of genotype on body mass (WT mice, 26.96 ± 0.21 g, n = 77; *Mc3r*^*TB/TB*^ mice, 29.93 ± 0.36 g, n = 79, p < 0.001). Unadjusted *ad libitum* food intake was similar between genotypes (WT, 2.95 ± 0.03 g/d, *Mc3r*^*TB/TB*^ mice, 2.95 ± 0.04 g/d). However, when including body mass and energy balance (weight gain or loss during the course of measurement) as covariates, *Mc3r*^*TB/TB*^ mice consume 6% fewer calories (2.86 ± 0.03 vs. 3.04 ± 0.03 g/d, p < 0.001).

A portion of the WT (n = 48) and *Mc3r*^TB/TB^ mice (n = 49) were then assigned to HFS and provided 2.20 ± 0.02 g/d of food (70–75% of their habitual calorie intake) at ZT4; food remaining in the hopper was weighed at ZT8 on day 2, 4 and 6 to estimate speed of food consumption. After 6 days of HFS, WT mice consumed 100% of food provided within 4 h of food presentation. In contrast, *Mc3r*^*TB/TB*^ mice had only consumed a portion (58.72 ± 3.94%) of the food after 4 h. This did not affect weight loss during the HFS (weight loss in g for WT, 2.88 ± 0.16 g; *Mc3r*^*TB/TB*^ mice, 2.83 ± 0.13 g) as food remaining in cages housing *Mc3r*^*TB/TB*^ mice (0.91 ± 0.09 g) was returned and consumed later.

**Figure 2:**
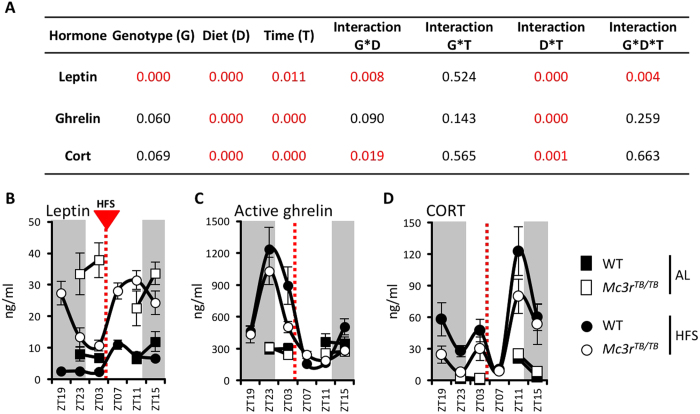
Neuroendocrine responses to HFS. *P* values for the effects of genotype (G), diet condition (D) and zeitgeber time (T) and their interactions determined by univariate analysis **(A)**. Significant P values are in red. Leptin **(B)**, acyl-ghrelin **(C)**, and corticosterone (CORT) **(D)** plasma levels at different time points during AL (square) or HFS (circle) conditions (n = 7–8). The dotted lines represent the time of meal presentation under HFS; dark period is indicated by grey shading. Black circles = WT mice; White circles = *Mc3r*^*TB/TB*^ mice. Note that for the AL condition, samples were not collected at ZT19 and ZT7.

As predicted, HFS had a significant effect on all three hormones measured (P < 0.001), with a significant effect of time of day (p < 0.05 for leptin, p < 0.001 for acyl-ghrelin and corticosterone, or CORT) and interaction between diet (HFS or ad libitum) and time of day (all p < 0.001) due to changes in feeding-related rhythms in circulating concentrations (Fig. 2A–D). HFS was associated with lower circulating leptin levels prior to the meal, with levels rising after food consumption (Fig. 2B). Acyl-ghrelin and CORT levels increased with HFS (Fig. 2C,D). Overall, these responses were retained in *Mc3r*^*TB/TB*^ mice (Fig. 2A–D). For leptin, there was however a significant interaction between all three variables (genotype, time and feeding condition) (p < 0.01). *Mc3r*^*TB/TB*^ mice are hyperleptinemic owing to obesity, however a decline in plasma leptin concentrations during HFS was still observed (Fig. 2A,B); meal-related increases were observed irrespective of genotype, but were more pronounced in *Mc3r*^*TB/TB*^ mice.

The interaction between all three variables (genotype, time and feeding condition) was not significant for acyl-ghrelin and CORT. Plasma acyl-ghrelin concentrations increased with HFS (diet effect, p < 0.001) at the time points preceding food presentation but were normal in the post-prandial period, irrespective of genotype (Fig. 2A,C). There was a strong trend for an effect of genotype (p = 0.06), perhaps due to lower levels pre-meal (Fig. 2A,C), and a significant effect of zeitgeber time (p < 0.001). However, irrespective of genotype acyl-ghrelin levels generally exhibited a clear peak at ZT23, and had begun to decline at ZT3 (1 h prior to food presentation) to normal levels.

HFS increased the amplitude of the daily rhythm of CORT, irrespective of genotype (Fig. 2A,D). For mice on HFS, a bimodal pattern of response was observed with a minor peak anticipating meal presentation and a second peak at the onset of the dark phase. While CORT levels began to increase in anticipation of mealtime, the peak may be an artifact induced by suppression following the meal at ZT4. There was a strong trend for an effect of genotype (p = 0.07), which may be due to lower CORT levels in *Mc3r*^*TB/TB*^ mice, particularly in the time points prior to feeding time (ZT23), and a highly significant effect of time (p < 0.001) due to the daily rhythm.

### Hypothalamic responses to HFS are attenuated in *Mc3r*-deficient mice

We previously published that hypothalamic *AgRP/Npy* expression is lower prior to meal time (ZT3) in *Mc3r*^*TB/TB*^ under HFS, and that hypothalamic *AgRP/Npy* expression correlates with FAA^10^. To further investigate meal-related responses to HFS, we measured expression of neuropeptides involved in appetite regulation expressed in ARC neurons (*AgRP, Npy, Pomc* and *Cart*) throughout the light/dark cycle in the mice used to assess neuroendocrine responses. We also examined genes linked to the stress response (*Fkb5, Cdkn1a, Nr3c1, Nr13c2*) and leptin signaling (*LepRb, Socs3*).

**Figure 3:**
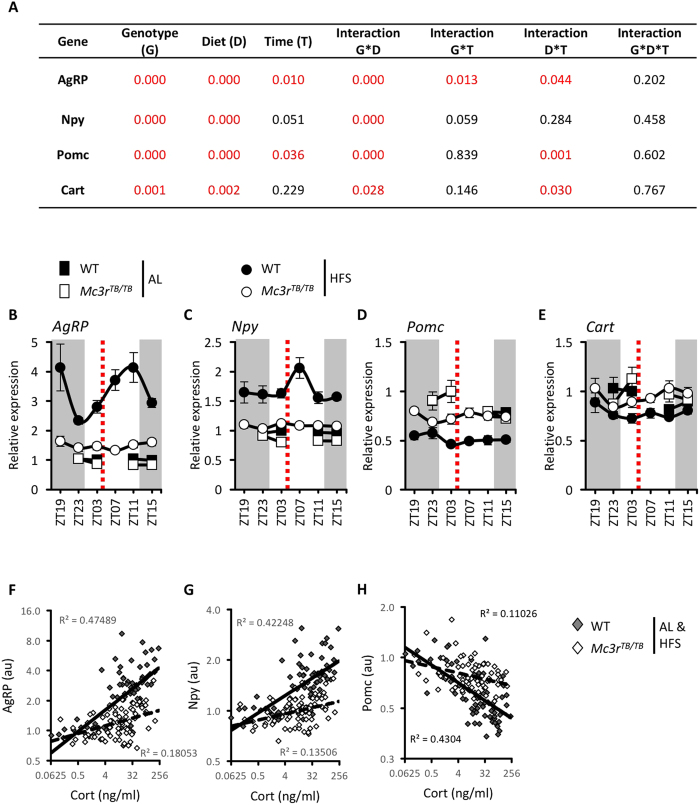
Response of hypothalamic neuropeptides involved in appetite control to HFS and their relationship with plasma corticosterone. Table representing for each gene studied P values of multiple way ANOVA for genotype (G), diet condition (HFS or *ad libitum*, D) and zeitgeber time (T) and their interactions **(A)**. Significant P values are in red. Relative expression of *AgRP* **(B)***, Npy* **(C)**, *Pomc* **(D),** and *Cart* mRNA **(E)** at different time points during *ad libitum* (AL, square) or hypocaloric feeding schedule (HFS, circle) conditions (n = 7–8). The dotted lines represent the time of meal presentation under HFS; dark period is indicated by grey shading. Black circles = WT mice; White circles = *Mc3r*^*TB/TB*^ mice. Note that for the AL condition, samples were not collected at ZT19 and ZT7. Scatterplots showing associations between plasma corticosterone (CORT) and hypothalamic *AgRP* **(F),** *Npy* **(G)** and Pomc **(H)** relative expression for WT and *Mc3r*^*TB/TB*^ mice. Dark grey circles = WT mice; White circles = *Mc3r*^*TB/TB*^ mice. Data shown are pooled from samples collected in the AL and HFS condition.

As expected, HFS markedly increased expression of *AgRP* and *Npy* mRNA in the hypothalamus in WT mice (Fig. 3A–C). HFS also reduced expression of *Pomc* and *Cart* mRNA encoding anorexigenic neuropeptides in WT mice (Fig. 3A,D–E). In *Mc3r*^*TB/TB*^ mice, *AgRP* and *Npy* expression were not responsive to HFS (Fig. 3A,B), while suppression of *Pomc* and *Cart* mRNA were attenuated (Fig. 3D,E).

This experiment also allows for an analysis of meal-related responses of hypothalamic gene expression. Surprisingly, *AgRP* expression was not suppressed by feeding, with peak expression occurring after meal at the same time as CORT (ZT11, compare Fig. 2D and 3B). Indeed, of the three hormones measured, CORT showed the strongest association with hypothalamic gene expression (Suppl. materials Table 1). A highly significant strong positive association was observed between plasma CORT and hypothalamic *AgRP* and *Npy* expression in WT mice (Suppl. materials Table 1, Fig. 3F,G), there was also a weak negative association between plasma CORT and hypothalamic *Pomc* mRNA (Suppl. materials Table 1, Fig. 3H). While still significant, the associations between CORT and *AgRP, Npy* and *Pomc* expression were weakened in *Mc3r*^*TB/TB*^ mice (Suppl. materials Table 1, Fig. 3F–H).

Genotype did not affect *LepRb* expression (Suppl. materials Fig. S1A,B). *Socs3* expression was slightly increased in *Mc3r*^*TB/TB*^ mice, consistent with hyperleptinemia (Fig. S1A–C). No major differences were also observed in hypothalamic *Nr3c1* (glucocorticoid receptor, Suppl. materials Fig. S1A–D), or *Nr3c2* (mineralocorticoid receptor; Suppl. materials Fig. S1A–E).

**Figure 4:**
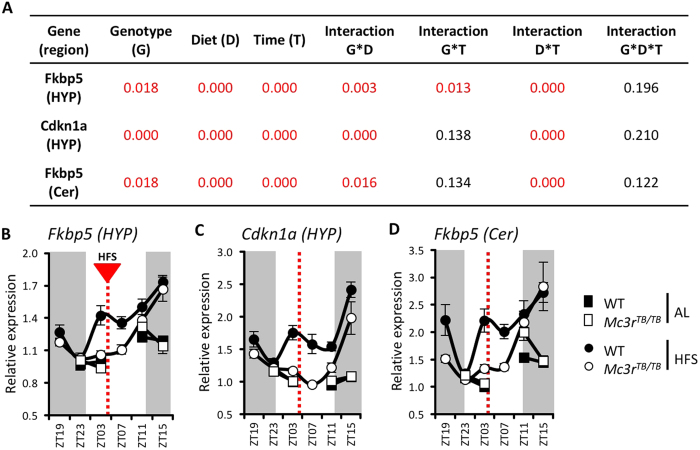
Expression of genes responsive to CORT in WT and *Mc3r*^*TB/TB*^ mice in response to HFS. Table representing for each gene studied P values of multiple way ANOVA for genotype (G), diet condition (D) and zeitgeber time (T) and their interactions **(A)**. Significant P values are in red. Relative expression of *Fkbp5* **(B)** and *Cdkn1a* in hypothalamus (HYP) **(C)**, and *Fkbp5* mRNA in the cerebellum (Cer) **(D**) at different time points (n = 7–8/group). The dotted lines represent the time of meal presentation under HFS; dark period is indicated by grey shading. Black circles = WT mice; White circles = *Mc3r*^*TB/TB*^ mice. Note that for the AL condition, samples were not collected at ZT19 and ZT7.

*Fkbp5* and *Cdkn1a* are stress responsive genes regulated during food restriction^39^. Daily profiles in *Fkbp5* and *Cdkn1a* expression were similar (Fig. 4A–C), and correlated strongly with plasma CORT concentrations (compare Figs 2D with 4B and C; Suppl. materials Table 1). During HFS, WT mice exhibited evidence of two peaks in *Cdkn1a* and *Fkbp5* expression coinciding with peaks in CORT; a major peak at ZT15 during the dark period and minor peaks at ZT3 anticipating food presentation (Fig. 4B,C). In *Mc3r*^*TB/TB*^ mice, peaks anticipating meal presentation was absent (Fig. 4B-C), correlating with a lower nadir and weakened pre-meal increase in circulating CORT (cf. Fig. 2D). An identical pattern of changes associated with genotype and feeding condition was observed for *Fkbp5* in the cerebellum, suggesting systemic failure (Fig. 4A,E).

To rule out compromised glucocorticoid signaling in *Mc3r*^*TB/TB*^ mice, we performed an additional study. *Mc3r*^*TB/TB*^ mice and littermate control were treated with dexamethasone (DEX, 10 mg/kg ip.) at ZT3 and then euthanized at ZT7. Genotype did not significantly affect treatment induced weight loss (Suppl. materials Fig. S2A) or stimulation of glucocorticoid-regulated genes (Suppl. materials Fig. S2B–E). DEX treatment modestly but significantly increased *AgRP* mRNA, irrespective of genotype (Suppl. materials Fig. S2F). No treatment effects were observed for *Npy, Pomc* or *Cart* expression (Suppl. materials Fig. 2F).

As anticipatory responses to HFS exhibits a circadian component^19^, we also examined expression of core clock and clock-output genes. No major effects of feeding condition, or interactions between feeding condition and genotype were observed in the hypothalamus, although were significant effects of feeding condition on *Bmal1* and *Npas2* expression and of genotype on *Npas2* and *Nr1d1* expression (Suppl. materials Fig. S3A–D,I). The presence of multiple oscillators in the hypothalamus may have confounded this analysis^40^; we therefore analyzed clock gene expression in the cerebellum, a region of the brain linked to motor control that harbors a food-entrainable oscillator^41^. There was a significant interaction between genotype, feeding condition and time (Suppl. materials Fig. S3E–I). Interestingly, only *Per2* was clearly affected by genotype around meal-time, with WT but not *Mc3r*^*TB/TB*^ mice exhibiting an increase anticipating food presentation (Suppl. materials Fig. S3H–I).

Altogether, the neuroendocrine response to HFS does not appear to be markedly compromised by loss of MC3Rs. In contrast, meal-related patterns in the expression of hypothalamic neuropeptides involved in regulating appetite are clearly disturbed in *Mc3r*^*TB/TB*^ mice. While the expression of stress-regulated genes in *Mc3r*^*TB/TB*^ mice appears overall to be responsive to HFS, there is evidence for a specific meal-related event that is Mc3r-dependent. Our findings thus suggest loss of MC3R-signaling causes a hypothalamic defect in the capacity to respond to fluctuations of circulating signals of energy status. Finally, the response of *AgRP* and *Npy* expression to HFS differs suggesting independent regulation. It is also worth noting that food intake did not suppress *AgRP* expression (Fig. 3B), with expression continuing to rise after the meal in line with CORT (Fig. 2D).

### Dissociation of feeding-related and nutrient-partitioning roles of MC3Rs

**Figure 5:**
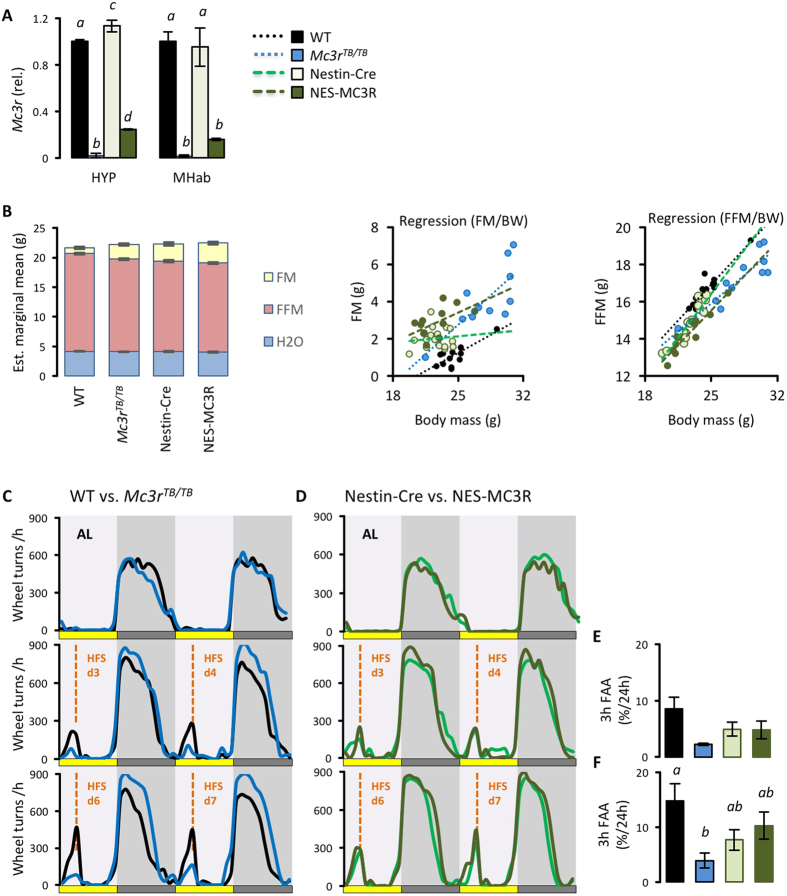
Restoring CNS Mc3r expression in *Mc3r*^*TB/TB*^ mice using nestin-Cre rescues FAA. **(A)** Assessment of *Mc3r* transcription in the hypothalamus (HYP) and medial habenula (MHab) of NES-MC3R mice. **(B)** Body composition showing averaged values by genotype (*left* panel) and scatterplots showing the relationship between fat mass (FM, *center* panel) and fat-free mass (FFM, *right* panel) and total body mass. **(C)** Wheel running data for WT (black) and *Mc3r*^*TB/TB*^ (blue) mice fed *ad libitum*, and then after 3, 4, 6 or 7d of hypocaloric feeding. **(D)** Wheel running data for Nestin-Cre (green) and NES-MC3R (red) mice fed *ad libitum* (AL) and then after 3, 4, 6 or 7d of hypocaloric feeding (HFS). (**E)** Averaged FAA data on days 3 and 4 of HFS; **(F)** Averaged FAA data on days 6 and 7 of HFS. In (**A**,**B**,**E** and **F**) columns not sharing letters are significantly different, p < 0.05.

We next examined whether neural MC3Rs are sufficient to restore normal appetitive responses during HFS. We first used nestin-Cre to broadly target neural transcription in *Mc3r*^*TB/TB*^ mice (NES-MC3R), as previously described^8^. While the nestin-Cre strain is not an ideal tool for selectively targeting the CNS^42^, we have observed strong Cre activity relative to another Cre strain targeting the CNS (synapsin-Cre) when using reporter mice (Girardet and Butler, unpublished observations). We therefore used it in proof-of-principle studies to determine whether rescuing *Mc3r* transcription in the brain would restore FAA. Analysis of *Mc3r* expression in the hypothalamus and medial habenula (MHab) indicated partial rescue in NES-MC3R relative to controls (Fig. 5A).

Nestin-Cre mice exhibit a mild hypopituitarism phenotype with reduced growth hormone secretion and altered body mass^42^. Body composition data are shown in Fig. 5B. In our laboratory condition, nestin-Cre mice exhibit a nutrient partitioning phenotype (FM for nestin-Cre negative, 1.74 ± 0.13 g; nestin-Cre positive, 3.13 ± 0.16 g; p < 0.001; FFM for nestin-Cre negative,16.07 ± 0.08 g; nestin-Cre positive, 15.17 ± 0.10 g, p < 0.001; values are estimated marginal means adjusted for total body mass, n = 33 for nestin-Cre negative;*Mc3r*^*TB/TB*^, n = 23 for nestin-Cre positive;*Mc3r*^*TB/TB or wt/wt*^). The anticipated effect of Mc3r-deficiency on adiposity was also observed (FM for *Mc3r*^*wt/wt*^, 1.95 ± 0.18 g; *Mc3r*^*TB/TB*^, 2.93 ± 0.16 g; p < 0.01; FFM for *Mc3r*^*wt/wt*^,15.88 ± 0.12 g; *Mc3r^TB/TB^*, 15.35 ± 0.10 g, p < 0.01; n = 25 for nestin-Cre (+ve or −ve); *Mc3r*^*wt/wt*^; n = 31 for nestin-Cre (+ve or −ve); *Mc3r*^*TB/TB*^). For both FM and FFM, there was a significant interaction between nestin-Cre and *Mc3r* genotype (P < 0.05), with the predicted nutrient partitioning phenotype associated with *Mc3r*-deficiency observed in nestin-Cre (−ve) but not in nestin-Cre (+ve) mice (Fig. 5B).

Comparison of FAA in “WT”, which are nestin-Cre (−ve) mice with normal *Mc3r* expression, and *Mc3r*^*TB/TB*^ mice shows the previously observed attenuated response (Fig. 5C,E,F). However, comparison of nestin-Cre positive and NES-MC3R mice shows no difference (Fig. 5D,E,F). FAA thus appears to be normalized in NES-MC3R mice compared to nestin-Cre controls (Fig. 5D,F) that are matched for adiposity, suggesting the role of MC3Rs in regulating food-seeking behaviors is dissociated from, and independent of, effects on adiposity and nutrient-partitioning.

### MC3R expressed in Nkx2.1(+ve) neurons are sufficient for FAA and hypothalamic responses during HFS

**Figure 6:**
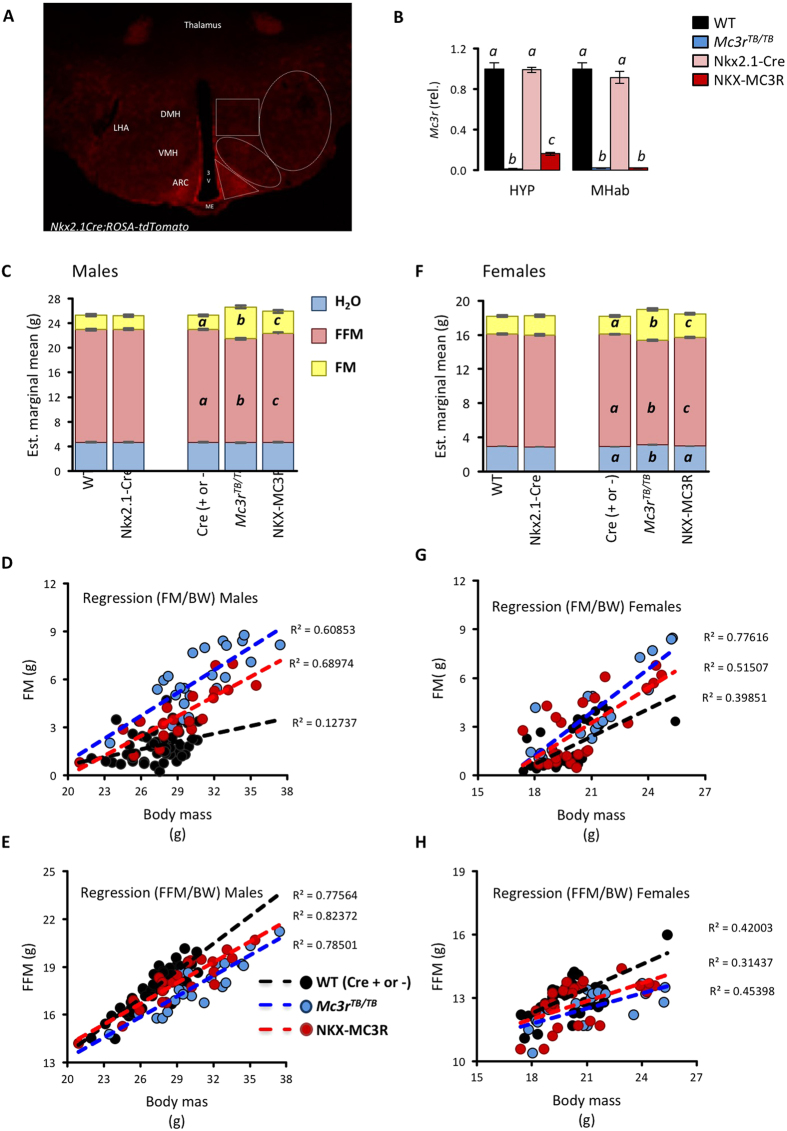
MC3Rs expressed in Nkx2.1(+ve) neurons have a modest influence on body composition. **(A)** Confocal image obtained from a reporter *Nkx2.1Cre; ROSA-tdTomato* mouse showing Nkx2.1-expressing cells at the level of the mediobasal hypothalamus. (**B**) Assessment of hypothalamic *Mc3r* expression by qRT-PCR revealed a recombination efficiency of 16% of Nkx2.1-Cre, but no rescue in the MHab. **(C–H)** Regression analysis comparing body composition in males (**C–E**) and females (**F–H**). Body composition (FM, FFM and H_2_O content) adjusted for total body mass are shown for males (**C**) and females (**F**). The Nkx2.1-Cre transgenic mice had normal values, and were therefore combined into one control group (Cre+ or −); values not sharing letters are significantly different, p < 0.01 for FM, FFM, p < 0.05 for H_2_O. Scatterplots showing the impact of genotype on relationship between body mass and FM (**D,G**) or FFM (**E,H**) are shown for males (**D,E**) and females (**G,H**). In (**B**,**C** and **F**) columns not sharing letters are significantly different, p < 0.05.

Nkx2.1-Cre targets transcription in regions of the forebrain and hypothalamus^28^,^29^. We confirmed Cre activation in our laboratory condition using an inducible tdTomato reporter mouse (Fig. 6A). Using qRT-PCR, rescue of *Mc3r* transcription was observed in the hypothalamus of nkx2.1-Cre;*Mc3r^TB/TB^* (NKX-MX3R) mice at levels comparable to that observed for nestin-Cre, with no rescue in the MHab (Fig. 6B). Regression analysis of body composition data shows the anticipated preferential deposition of FM over FFM when expressed as estimated marginal means adjusted for body mass in *Mc3r*^*TB/TB*^ mice (Fig. 6C–H). NKX-MC3R mice exhibited a modest rescue of the nutrient partitioning phenotype associated with *Mc3r*-deficiency in males (Fig. 6C–E) and females (Fig. 6F–H). NKX-MC3R mice accumulated 1–2 g less FM as a function of total body mass compared to *Mc3r*^*TB/TB*^ mice, irrespective of sex (Fig. 6D,G). In males and female mice, the impact of MC3Rs expressed in Nkx2.1(+ve) neurons on normalizing FFM was also modest (Fig. 6E,H).

**Figure 7:**
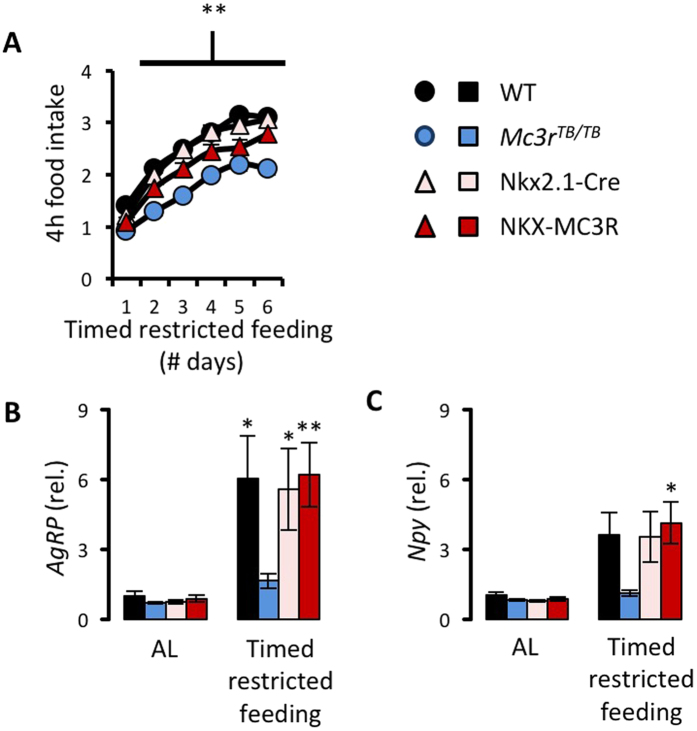
Hypothalamic MC3Rs are sufficient to restore caloric loading and response of AgRP/Npy neurons during restricted feeding. Food intake in the 4 h period following time-restricted feeding (TRF) is compared **(A)** Hypothalamic expression (relative to WT controls fed ad libitum) is shown for *AgRP* (**B**) and *Npy* (**C**) in female mice fed *ad libitum* (AL) or after 6 d of restricted feeding (n = 4–6/group). Restricted feeding significantly increased expression of *AgRP* and *Npy* in WT, Nkx2.1-Cre and NKX-MC3R mice, but not in *Mc3r*^*TB/TB*^ mice. In A, *p < 0.05 compared to controls (WT vs. *Mc3r^TB/TB^*). In (**B** and **C**) *p < 0.05 compared to AL condition; **p < 0.01 compared to AL condition.

**Figure 8:**
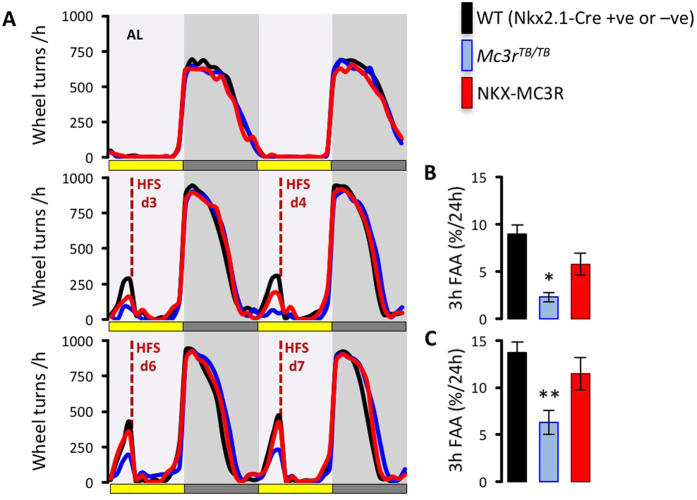
Hypothalamic MC3Rs are sufficient to restore expression of FAA in *Mc3r*^*TB/TB*^ mice. **(A)** Wheel running data is shown for controls, *Mc3r*^*TB/TB*^ and NKX-MC3R mice at baseline in ad libitum condition (AL), and after 3, 4, 6 and 7 days of HFS (mice fed at ZT4). Averaged FAA data for days 3 and 4 **(B)**, and days 6 and 7 **(C)**, are shown. The FAA data shown in B and C are activity in the 3 h window prior to food presentation (ZT1-ZT4), and are adjusted for 24 h activity. (*Mc3r*^*TB/TB*^ mice, n = 20; NKX-MC3R mice, n = 19; controls, n = 38). Controls are pooled WT and Nkx2.1-Cre mice which exhibited similar FAA responses. *p < 0.05 compared to controls; **p < 0.01 compared to controls, p < 0.05 compared to NKX-MC3R mice.

We next compared responses of NKX-MC3R and *Mc3r*^*TB/TB*^ to situations of negative energy balance. Measurement of 4 h food intake during time restricted feeding revealed lower intake in *Mc3r*^*TB/TB*^ mice compared to WT controls (Fig. 7A); this difference was not observed when NKX-MC3R mice were compared to Nkx2.1-Cre mice (Fig. 7B). An analysis of hypothalamic gene expression in mice fed *ad libitum* or subject to HFS for 6d indicated that the response of AgRP/Npy neurons was restored in NKX-MC3R mice (Fig. 7C,D). In this experiment, there was no significant effect of timed restricted feeding or genotype on *Pomc* expression (data not shown), perhaps due to differences in feeding protocol and/or sex (females were used for this experiment versus males used for the experiment shown in Fig. 3). Finally, analysis of FAA indicated impaired behavioral adaptation to daytime feeding in *Mc3r*^*TB/TB*^ mice was rescued in NKX-MC3R mice (Fig. 8A–C). These results suggest that MC3Rs expressed in Nkx2.1(+ve) neurons are sufficient to restore the response of *AgRP/Npy* gene expression to HFS and for expression of FAA.

## Discussion

These studies provide important new information on the importance of MC3R signaling in driving homeostatic appetitive responses to hypocaloric conditioning. It is well known that weight loss is associated with homeostatic responses involving increased appetite. The current results confirm that intact MC3R signaling is crucial for compulsive behavioral responses involving consumption of large meals in situations of prolonged negative energy balance. This phenotype may be at least partially explained by the failure of crucial “fasting-responsive” neurons in the ARC to respond to internal cues of metabolic condition.

While the analysis of hypothalamic expression of orexigenic neuropeptides is not novel, the data nevertheless reinforce previous observations by our laboratory^10^ and others^12^. Moreover, combining a rigorous analysis of daily patterns and meal effects in neuroendocrine stress-related hormones and hypothalamic expression of stress-responsive genes, provides novel insights into the relationship between CORT and stress-responsive genes in the nervous system. Finally, the observation that MC3Rs expressed in Nkx2.1(+ve) neurons are sufficient to restore appetitive responses to hypocaloric conditioning is novel, and highly significant. This important data further refines our understanding of how MC3R signaling neurons may exert strong regulatory control over local and peripheral nutrient sensor signals on the cluster of fasting-responsive neurons that govern appetite.

Feeding protocols limiting caloric intake are a severe metabolic stressor for mice^43^,^44^. Most food consumption occurs within 1 h of food presentation; mice therefore adapt to repetitive cycles of gorging interrupted by prolonged fasting periods (>20 h)^9^,^44^. The gorging response is minimally affected by the method used to restrict calories. It is observed when food access is restricted to 4 h windows each day using mechanical barriers^9^, or when a hypocaloric meal is placed at 24 h intervals in food-hoppers^44^. An examination of meal structure (Fig. 1) clearly demonstrates the abnormal behavioral response of *Mc3r*-deficient mice to HFS. The predicted gorging behavior observed in control mice involves a progressive adaptation of feeding strategy leading to fewer larger meals. When populations are competing for limited nutrient supply, this response could be considered as adaptive. That this response is clearly and markedly attenuated in *Mc3r*-deficient mice provides further evidence of a crucial role for this receptor in the expression of altered feeding behavior (appetite) in hypocaloric situations.

The literature on the impact of *Mc3r*-deficiency on feeding behavior when nutrients are not limiting is ambiguous. Various groups have reported hypophagia^38^, normal intake^8^,^9^,^37^ or even modest hyperphagia^15^,^36^ in *Mc3r*-deficient models, suggesting sensitivity to background strain and environment. Here we presented data from two experiments in the *ad libitum* condition. The data from the largest study reported to date (N = 77–79/group) suggest a modest reduction in *Mc3r*-deficient mice when caloric intake is adjusted using body mass and energy balance as covariates. The result showing reduced frequency of bouts assessed using an automated system in the *ad libitum* condition is novel, and may indicate reduced interest in food.

The significance of these results for the human condition is unclear given the rarity of *MC3R* mutations inducing loss of function in the general population. However, two studies reported a “slowness-in eating” phenotype with *MC3R* mutations that alter signaling properties in cultured cells^17^,^18^. Our results suggest that the feeding phenotype associated with loss of MC3R signaling in mice is amplified with hypocaloric conditioning. Whether *MC3R* haploinsufficiency in humans would similarly affect appetite during situations of prolonged hypocaloric conditioning has not been explored. Our data also raise interesting questions about how selective MC3R compounds would affect feeding behavior.

Appetitive responses to weight loss can limit the effectiveness of voluntary reduction in caloric intake as an obesity therapy. The finding that rescuing *Mc3r* expression in Nkx2.1(+ve) neurons restores compulsive behavioral responses involving meal anticipation and consumption of large meals is therefore significant. Multiple populations of neurons are involved in the activation of motivational responses, with fasting responsive AgRP neurons forming a focal point for local (synaptic and nutrient sensing) and humoral inputs from the periphery^45^,^46^,^47^. These observations suggest a population of Nkx2.1(+ve);Mc3r(+ve) neurons that are instrumental in integrating multiple signals of metabolic condition to enhance appetite during hypocaloric situations.

Cre activity in Nkx2.1-Cre mice has been mapped to GABAergic, Npy^+ve^, Pomc^+ve^ and TH^+ve^ neurons in the ARC and glutamatergic neurons in the ventromedial hypothalamus (VMH)^29^. It is however important to note that Cre activity in this model is not restricted to the hypothalamus. Cre activity has been observed throughout the telencephalon, including the cerebral cortex, amygdala, olfactory bulb, striatum, globus pallidus, septum, nucleus basalis and hippocampus^28^. *Mc3r* expression has been observed in the amygdala, preoptic area and hippocampus^27^. While it is possible that MC3Rs expressed in hypothalamic nuclei support appetitive responses to hypocaloric conditions, further studies are required to identify which subset of MC3R(+ve);Nkx2.1(+ve) neurons is responsible for restoring appetite responses. MC3Rs expressed by steroidogenic factor-1 (SF1) neurons in the ventromedial hypothalamus appear to be insufficient to restore FAA^8^. Further investigation of the network of hypothalamic Nkx2.1(+ve);Mc3r(+ve) neurons populations is warranted.

Another limitation to the current study is that the examination of hypothalamic responses was limited to gene expression, which may not necessarily correlate with changes in neural activity^48^. Further studies assessing neural activity using other techniques are therefore needed. It is also unclear whether Nkx2.1(+ve);Mc3r(+ve) neurons are actively involved in the daily processing of metabolic cues, or affect maturation of hypothalamic circuits governing appetite.

Interestingly, while MC3Rs expressed in Nkx2.1(+ve) neurons appear to be linked strongly to neural circuits governing appetite control, they appear insufficient to restore normal nutrient partitioning. Based on the current results and previously published observations^8^,^14^, the identity and site of action for MC3Rs involved in regulating nutrient partitioning remains unclear.

Other significant advances provided by these experiments stem from the analysis of the neuroendocrine response to HFS. Neuroendocrine hormones and gut peptides exhibiting anticipatory responses to meal-time may have a role in preparing the nervous system for nutrient consumption, enhancing feeding efficiency when nutrients are limiting^49^,^50^,^51^. With HFS, acyl-ghrelin and CORT exhibited increased amplitude in the 24 h rhythm (Fig. 2). For acyl-ghrelin, the peak anticipates mealtime irrespective of genotype. This suggests that entrainment of acyl-ghrelin released to anticipate mealtime in response to food-entrainable oscillators in X/A-like cells of the stomach^52^ is retained in *Mc3r*^*TB/TB*^ mice.

The impact of genotype and HFS on plasma leptin concentrations was predictable. As reported^35^,^38^,^53^, obesity associated with *Mc3r*-deficiency mice causes hyperleptinemia. HFS reduced plasma leptin concentrations during the premeal phase, with a marked postprandial increase observed irrespective of genotype. While plasma leptin concentrations in *Mc3r*-deficient mice were markedly reduced in the period leading up to food presentation (ZT23 and ZT3), the level did not fall below the values for controls fed *ad libitum*. This observation potentially confounds our interpretation of studies examining behavioral and metabolic adaptation in *Mc3r*-deficient mice. The decline in leptin is a powerful signal driving behavioral and metabolic adaptation to weight loss^1^,^2^. It is thus feasible that failure of leptin to fall below a threshold level may contribute to the phenotype observed in these experiments. However, the results from the NES-MC3R experiment suggest that the loss of food anticipatory behaviors associated with *Mc3r*-deficiency is not secondary to obesity, as FAA is restored independently of reductions in FM. Moreover, as shown in Fig. 5, NKX-MC3R mice exhibit only modest improvements in adiposity but have marked improvement in food anticipatory behaviors, gorging behavior and responsiveness of AgRP/Npy to signals of altered metabolic condition.

For CORT and the hypothalamo-pituitary-adrenal (HPA) axis, interpreting the interaction between genotype and feeding condition is more complex. While there was a marked increase in amplitude, the retention of the peak at ZT11 marking the transition between light and dark periods is surprising, as we anticipated a phase shift with peak levels occurring pre-meal based on the literature^49^. This discrepancy could be due to insufficient duration of HFS to produce a phase shift in the control of adrenal steroidogenesis by circadian oscillators^54^. However, work by other laboratories indicated that peripheral clocks adjust with 7d of restricted feeding^55^. Furthermore, the daily pattern of CORT fluctuations under restricted feeding also depends on the interval between meal presentation and activity onset and the severity of the caloric restriction^56^,^57^.

The current study consolidates evidence for dissociation between circadian oscillator-controlled stress responses responsive to light- and calorie-dependent inputs^58^,^59^. The response of glucocorticoid-regulated “stress response” genes in the hypothalamus and cerebellum suggests HFS has induced a peak in the activity of stress-regulated genes that anticipates food presentation. The response is likely driven by or responsive to systemic non-local inputs, as we could not detect *Mc3r* expression in the cerebellum, consistent with other reports^5^. The absence of the feeding-related peak in the hypothalamus and cerebellum of *Mc3r*-deficient mice suggests an independent stress-response governed by nutrient availability that is dependent on MC3R signaling. The second peak entrained to the transition between the light- and dark-phases is retained in *Mc3r-*deficient mice, and likely represents the light-entrained rhythm in adrenal steroidogenesis^54^.

Renquist *et al*. reported that the response of the HPA axis to a single bout of fasting is dependent on functional MC3Rs, however the response to negative energy balance associated with prolonged bouts of restricted feeding is maintained^12^. While our data are consistent with Renquist’s data, it differs in suggesting that a component of the stress response is attenuated in *Mc3r-*deficient mice. The timing of a meal-entrained stress response, indicated by peaks in expression of stress-responsive genes, coinciding with nutrient consumption is dependent on functional MC3Rs. This response is likely indirect and may involve a circadian component. Rhythms in the expression of core elements and circadian oscillator output genes that show entrainment in control mice in the cerebellum do not respond in *Mc3r-*deficient mice (Suppl. Fig. 2).

In summary, the results of these experiments further support the crucial role of MC3R signaling in the expression of homeostatic adaptive behavioral responses to hypocaloric conditioning. Rescuing *Mc3r* expression in the hypothalamic and limbic structures improves appetitive responses to internal cues of metabolic condition, but at best has a minor inhibitory effect on adiposity. The primary site(s) and mechanisms of action of MC3Rs involved in nutrient partitioning therefore remain unclear. One interpretation of the results from studies of the hypothalamic and behavior responses to HFS is that critical fasting-responsive melanocortin neurons in the hypothalamus of *Mc3r*-deficient mice are at least partially uncoupled from signals of metabolic condition. However, caution must be applied as this interpretation is based exclusively on gene expression data that may not necessarily correlate with activity of ARC neurons^48^. The basis of the behavioral phenotype could involve ‘active’ regulatory functions of MC3Rs in setting the gain in the response of fasting-responsive neurons to signals of metabolic condition. Alternatively, MC3R signaling in Nkx2.1(+ve) neurons may be crucial for the development and maturation of hypothalamic circuitry involved in appetite control. When combined with recently published data^14^, MC3Rs expressed in hypothalamic and limbic neurons overall are suggested to have functions that promote feeding behavior and maintain behavioral flexibility in response to internal cues that control incentive salience and motivational state. Finally, the current studies further dissociate the paradoxical impact of *Mc3r-*deficiency to reduce feeding behavior while regulating nutrient partitioning to favor the development of an obese phenotype.

## Methods

### Animal husbandry

All of the experiments involving mice were performed in accordance to the guidelines and regulations provided by the Institutional Animal Care and Use Committees of the Scripps Research Institute and Saint Louis University School of Medicine, which reviewed and approved the studies.

The mice used in these studies were acclimated to single-housing at a constant temperature of 23C with food and water provided *ad libitum* under a 12:12 light-dark cycle, unless otherwise specified. In B6(Cg)-*Mc3r*^*tm1Butl*^/J (*Mc3r*^*TB/TB*^) mice, *Mc3r* transcription is inhibited by a loxP-flanked transcriptional blocker; these mice are primarily on the C57BL/6J background with a small contribution from/6N. The percentage of genes derived from C57BL/6J and/6N in each of our experimental mice is presently unknown. *Mc3r* transcription was rescued throughout the CNS (NES-MC3R mice) using B6.Cg-Tg(Nes-cre)1Kln/J (nes-cre)^60^ backcrossed on the *Mc3r*^*TB/TB*^ background. *Mc3r* transcription was rescued using Nkx2.1-Cre (C57BL/6J-Tg(Nkx2–1-cre)2Sand/J) (NKX-MC3R mice)^28^.

B6.Cg-*Gt(ROSA)26Sor*^*tm14(CAG-tdTomato)Hze*^/J was crossed with Nkx2.1-Cre to obtain Nkx2.1Cre;ROSA-tdTomato mice in order to visualize recombination pattern at the level of the mediobasal hypothalamus. All mice studied were littermates obtained from heterozygous breeding. Cre genotype was kept to heterozygosity and carried by the male for breeding purpose. Genotyping PCR using tail-tip DNA was used to assess germline recombination. Animals showing Cre-mediated recombination in the tail were then removed from the study. Note that for experiment involving Cre-mediated excision, Cre-positive mice were also studied in order to evaluate and account for the effect of Cre expression itself.

For consistency with previous experiments^7^,^8^,^9^,^10^,^11^,^14^,^61^,^62^, we used a refined low fat/high carbohydrate diet (LFD, 10% kcal from fat, 20% kcal/protein and 70% kcal/carbohydrates, D12450B; Research diets Inc., New Brunswick, NJ). Nuclear magnetic resonance (NMR, Bruker Minispec) was used to measure fat mass (FM), fat-free mass (FFM) and free H_2_O^14^.

### Hypocaloric conditioning protocol

To assess circadian- and feeding-related adaptation to scheduled feeding, a simple protocol was used. A single meal, amounting to 70–75% of normal habitual food intake determined empirically, was provided daily at ZT4 (ZT, zeitgeber time; ZT 0 and ZT12 represent times of dark/light and light/dark transition). After 4 hours, the amount of food of remained was weighed on days 2, 4 and 6 to estimate consumption. Any remaining food was returned to the cage.

Neuroendocrine and hypothalamic responses were examined using adult (12–14wk) male wild type (WT, n = 77) and *Mc3r*^TB/TB^ (n = 79) mice euthanized at 4 h intervals throughout the day with the HFS group receiving food. Red lights were used as needed to avoid disrupting daily rhythms.

Contribution of hypothalamic MC3Rs to hypothalamic responses was investigated in adult female mice (n = 4–6) euthanized at ZT3.

### Assessment of food intake and Meal pattern analysis

Meal structure was examined using an automated system for continuous monitoring of food consumption (BiodaQ 2.3, Research Diets Inc., New Brunswick, NJ) and BiodaQ 2.3 software. ‘Bouts’ indicate disturbance of the hopper and instability in scale readings suggesting approach and investigation; actual changes in food weight were used to estimate meal size. Meals were defined as bouts occurring within 5 minutes of each other and consumption of ≥0.02 g.

Mice acclimated to single housing on bedding with no caloric value (aspen chips) and LFD for 3 wk were transferred to BiodaQ cages. After 10d of acclimation, baseline feeding behavior was established using 2d of recordings before food deprivation.

Adult male mice (8–10 wk) were then subjected to HFS for 6d with food provided at ZT4 with water provided *ad libitum*.

Nkx2.1-Cre male and female mice (20 wk) were then submitted to a timed food restriction protocol with food access granted access for 4 h (from ZT4 to ZT8)^9^.

### Operant conditioning behavior

Self-administration experiments were performed as previously described using operant chambers (Coulbourn Instruments)^14^. Briefly, mice were trained in operant chambers housed in sound-attenuating cubicles (Coulbourn Instruments). Each chamber was equipped with two levers (predetermined to be “active” or “inactive”), a food pellet hopper and a house light. Prior to training, 3.5–4 months old male mice were subjected to 14 days of caloric restriction in order to decrease body weight to 85–90% of normal. Weight loss was then maintained throughout the study by feeding the mice a hypocaloric meal after the end of the training session. Mice were first habituated to the chambers with chocolate flavored food pellets (sucrose; Dustless Precision Pellets 20 mg, Bioserv) dropping every minute concomitantly with the activation of a light cue over the active lever. Following two days on this “magazine” schedule, mice were trained for 7 days in a Fixed Ratio 1 (FR1) schedule; each “active” lever pressing resulted in the delivery of a sucrose pellet and the activation of the light cue. Pressing the inactive lever had no outcome. The training session lasted 60 min with a 1 sec time out period following the delivery of the pellet. Two cohorts of WT and *Mc3r*^TB/TB^ male mice maintained either under a normal 12 h light:12 h dark cycle or an inverted 12 h dark:12 h light cycle were studied respectively during their inactive (light phase) and active (dark phase) period. Note that all manipulation of the cohort maintained under the inverted light-dark cycle was performed under red light.

### Assessment of locomotor activity

FAA was assessed using mice housed in cages with free access to running wheels as previously described^10^. Briefly, after a 1 wk acclimation period to wheel-equipped cages in a 12:12 light-dark (LD), food was measured 3 times over a 1 week period before all food was removed and mice were submitted to HFS protocol. Wheel running data was analyzed using Clocklab software (Actimetrics, Evanston, IL).

### Blood chemistries

Trunk blood was collected in the presence of AEBSF (1 mg/ml, Sigma, St Louis, MO). Plasma was collected after centrifugation (15 min, 2000 g, 4C). To measure acyl-ghrelin, a portion of plasma was acidified with HCl (0.05N). Mouse ELISA kits were used to measure acyl-ghrelin (Millipore, Billerica, MA), leptin (Crystal Chem, Downers Grove, IL) and corticosterone (CORT; Enzo Life Sciences Inc., Farmingdale, NY); intra- and the inter-assay CV were < 5%.

### Drug treatment

Mice acclimated to single-housing were injected with dexamethasone 21-phosphate disodium salt (10 mg/kg ip., D1159, Sigma, St Louis, MO) or saline at ZT3 and returned to home cages for 4 h before sacrifice.

### Gene expression analysis

The hypothalamus, medial habenula (MHab) and cerebellum were dissected from frozen brains on dry ice and total RNA extracted using Trizol (Invitrogen, Life Technologies). Total RNA was treated with a DNAfree kit (Ambion, Life Technologies) and 800 ng used for cDNA synthesis (Superscript III Reverse transcription kit, Invitrogen). Quantitative PCR was performed in 384-well plates using Taqman gene expression and QuantStudio 6 or 7 Detection Systems (Applied Biosystems, Life Technologies) with *Tbp* as the reference gene.

### Statistical analysis

All data are presented as mean ± SEM. Statistical analyses were performed using GraphPad Prism 6 and IBM SPSS Statistics software. The effect of genotype of body composition was assessed by ANCOVA with total body mass as a covariate; FM, FFM and free H_2_O are presented as estimated marginal means adjusted for total body mass unless stated otherwise^63^,^64^. Differences between two genotypes were assessed by unpaired *t* test. For feeding studies, effects of *Mc3r* genotype and time were analyzed using 2-way ANOVA with repeated measures followed by Bonferroni’s post hoc test. In experiments examining the effect of feeding and genotype on 24 h rhythms, multi-factor ANOVA were performed with feeding (HFS or ad libitum), genotype and time as independent variables. Spearman correlation was used to evaluate relationships between neuropeptide expression and hormonal levels. A *p* value < 0.05 was considered significant.

## Additional Information

**How to cite this article**: Girardet, C. *et al*. Melanocortin-3 receptors expressed in Nkx2.1(+ve) neurons are sufficient for controlling appetitive responses to hypocaloric conditioning. *Sci. Rep.* **7**, 44444; doi: 10.1038/srep44444 (2017).

**Publisher's note:** Springer Nature remains neutral with regard to jurisdictional claims in published maps and institutional affiliations.

## Supplementary Material

Supplementary Data (3 Figures, 1 Table)
