# Supplementary material for: Melanocortin-3 receptors expressed in Nkx2.1(+ve) neurons are sufficient for controlling appetitive responses to hypocaloric conditioning
Source: Sci Rep. 2017 Mar 15;7:44444. doi: 10.1038/srep44444 (PMC5353610; doi:10.1038/srep44444)
Supplement: Supplementary Data (3 Figures, 1 Table) [file srep44444-s1.pdf]

SUPPLEMENTARY INFORMATION

Melanocortin-3 receptors expressed in Nkx2.1(+ve) neurons are sufficient for controlling appetitive responses to hypocaloric conditioning.

Clémence Girardet[1], Maria M. Mavrikaki[1], Joseph R. Stevens[1], Courtney A. Miller[2], Daniel L. Marks[3] and Andrew A. Butler[1]

Author affiliations:

1. Department of Pharmacology & Physiology, Saint-Louis University, Saint-Louis, MO 63104.
2. Departments of Metabolism and Aging and Neuroscience, The Scripps Research Institute, Jupiter, FL 33458.
3. Papé Family Pediatric Research Institute, Oregon Health & Science University, Portland, OR 97239, USA.

Corresponding author and address for reprint requests:
Andrew A. Butler,
Department of Pharmacology & Physiology
Saint Louis University School of Medicine
1402 S Grand Blvd
St. Louis, MO 63104
Telephone: (314) 977-6425;
Fax: (314) 977-6410;
Email: Butleraa@slu.edu

A

Gene	Genotype (G)	Diet (D)	Time (T)	Interaction G*D	Interaction G*T	Interaction D*T	Interaction G*D*T
**ObRb**	0.338	0.008	0.000	0.480	0.867	0.001	0.698
**Socs3**	0.000	0.000	0.000	0.408	0.004	0.000	0.323
**Nr3c1**	0.032	0.062	0.008	0.648	0.765	0.292	0.818
**Nr3c2**	0.136	0.001	0.004	0.296	0.108	0.075	0.857

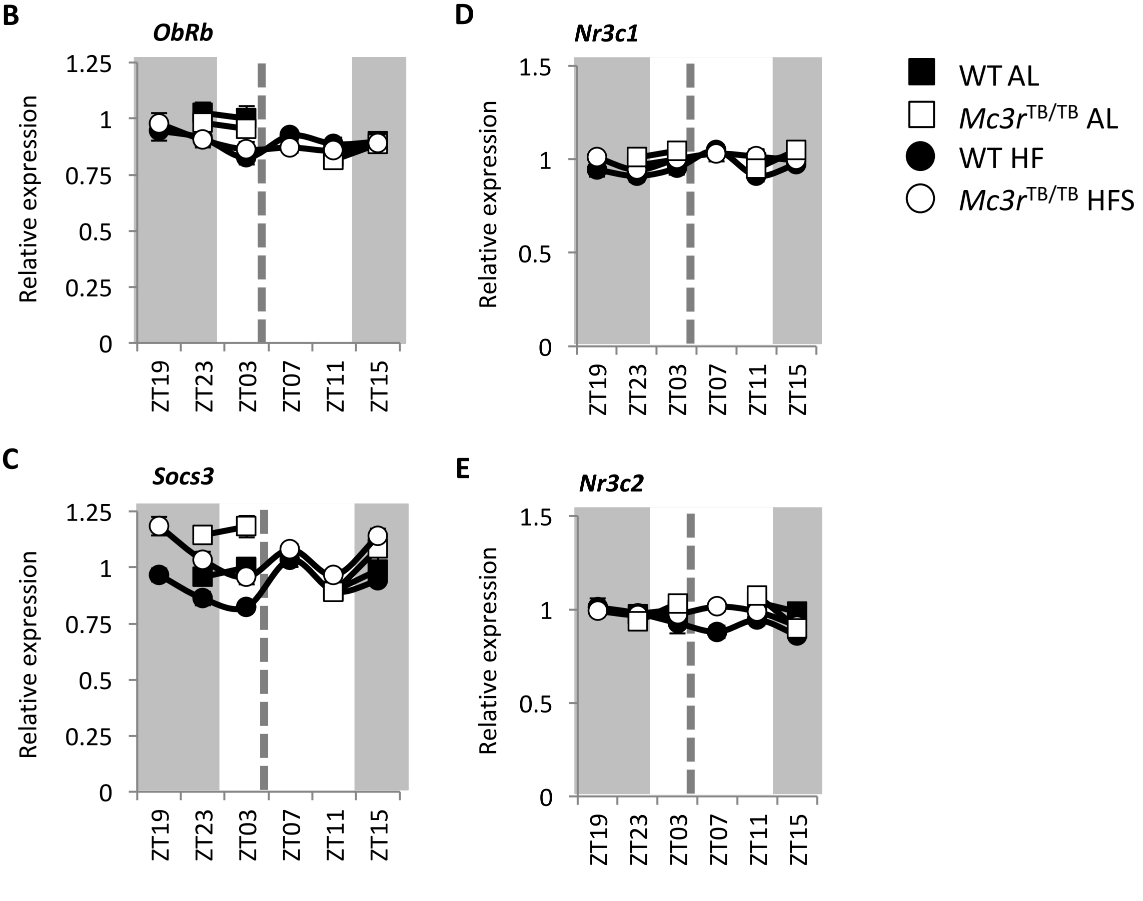

Online suppl. figure S1. Leptin and corticosterone responsive gene expression in the hypothalamus during *ad libitum* and response to 7 d hypocaloric feeding in WT & *Mc3r*$^{TB/TB}$ male mice.

(A) Table representing for each gene studied P values of multiple way ANOVA for genotype (G), diet condition (D) and zeitgeber time (T) and their interactions. Significant P values are in red. **(B)** Relative expression of *ObRb,* **(C)** *Socs3*, **(D)** *Nr3c1* and **(E)** *Nr3c2* mRNA at different time points during AL (square) or RF (circle) conditions (n=7-8). The dotted lines represent the time of meal presentation under HF. The shaded areas represent the dark period of the light-dark cycle.

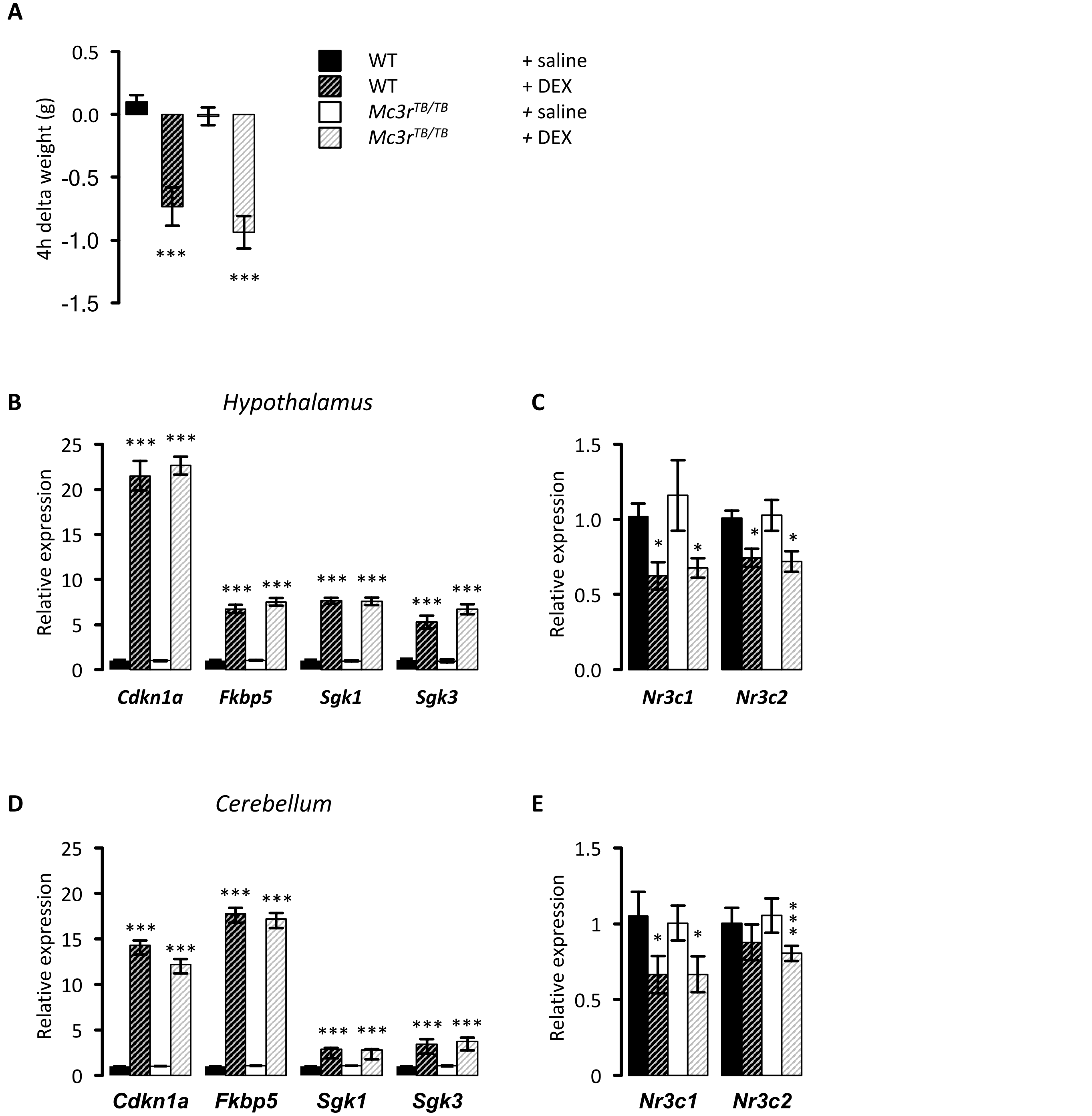

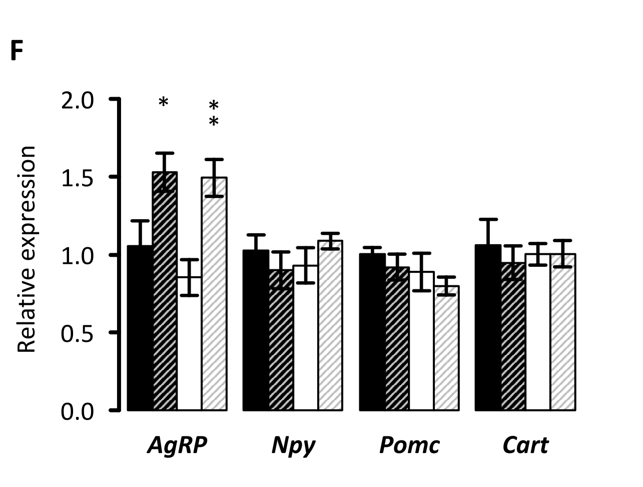

Online suppl. figure S2. Normal response of *Mc3r*$^{TB/TB}$ mice to dexamethasone injection.

(A) Body weight difference 4h post-treatment, **(B-E)** Corticoid responsive transcripts levels in the hypothalamus (B-C) and cerebellum (D-E) in WT (black) and *Mc3r*$^{TB/TB}$ (white) male mice treated at ZT3 with either saline (plain bars, n=6-7) or 10mg/kg dexamethasone (hatched bars, n=6-8). **(F)** Response of hypothalamic neuropeptides involved in feeding behavior.

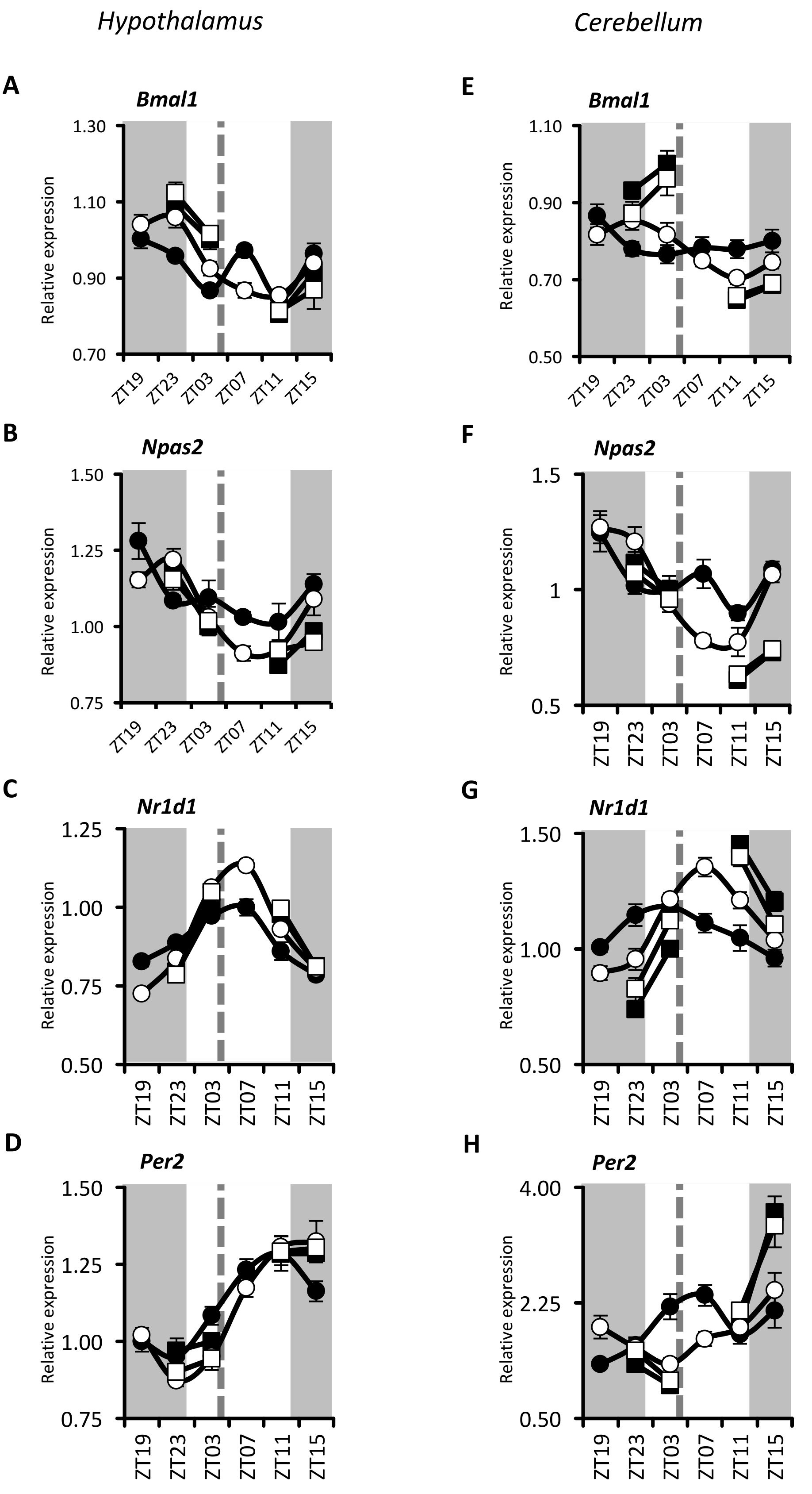

Online suppl. figure 3. Clock gene expression in the hypothalamus and cerebellum during *ad libitum* and response to 7 d hypocaloric feeding in WT & *Mc3r*$^{TB/TB}$ male mice.

(A-E) Relative expression of *Bmal1*, **(B-F)** *Npas2*, **(C-G)** *Nr1d1* and **(D-H)** *Per2* mRNA in the hypothalamus (A-D) and cerebellum (E-H) at different time points during AL (square) or RF (circle) conditions (n=7-8). The dotted lines represent the time of meal presentation under HF. The shaded areas represent the dark period of the light-dark cycle. **(I)** Table representing for each gene studied P values of multiple way ANOVA for genotype (G), diet condition (D) and zeitgeber time (T) and their interactions. Significant P values are in red.

I

Gene (region)	Genotype (G)	Diet (D)	Time (T)	Interaction G*D	Interaction G*T	Interaction D*T	Interaction G*D*T
**Bmal1 (HYP)**	0.393	0.002	0.000	0.316	0.000	0.000	0.373
**Npas2 (HYP)**	0.040	0.001	0.000	0.696	0.103	0.026	0.030
**Nr1d1 (HYP)**	0.035	0.272	0.000	0.362	0.000	0.000	0.324
**Per2 (HYP)**	0.256	0.765	0.000	0.600	0.003	0.232	0.167
**Bmal1 (Cer)**	0.199	0.015	0.000	0.219	0.650	0.000	0.014
**Npas2 (Cer)**	0.076	0.000	0.000	0.740	0.001	0.000	0.048
**Nr1d1 (Cer)**	0.148	0.951	0.000	0.804	0.000	0.000	0.000
**Per2 (Cer)**	0.987	0.075	0.000	0.303	0.001	0.000	0.019

Supplemental Table 1. Correlation coefficients (Spearman's rho) between neuroendocrine hormones and hypothalamic gene expression in WT and *Mc3r*$^{TB/TB}$ mice (n=77-79). * p<0.05, ** p<0.01. Overall, CORT exhibited the strongest and most consistent correlations with hypothalamic gene expression in control mice. The data shown are for all time points measured. Associations between CORT and hypothalamic genes were stronger with removal of ZT7 data (postprandial dip in CORT, Fig. 2C).

WT

	CORT	acyl-ghrelin	leptin	*agrp*	*npy*	*pomc*	*cart*	*ptpn11*	*obrb*	*socs3*	*fkbp5*	*crh*
**CORT**	1	.241*	-.257*	.660**	.585**	-.592**	-.512**	0.012	-.311**	-.408**	0.600**	-0.152
**Acyl-ghrelin**		1	-.557**	0.02	0.067	-0.065	-0.015	0.036	-0.053	-.329**	-0.094	0.056
**leptin**			1	-.277*	-.242*	0.211	0.174	0.207	0.108	.446**	0.07	-0.089
agrp				1	.875**	-.654**	-.418**	.226*	0.023	-0.082	.535**	0.062
npy					1	-.720**	-.506**	0.107	-0.099	-0.101	.507**	0.033
pomc						1	.568**	-0.053	.334**	.422**	-.539**	0.004
cart							1	0.211	.341**	.280*	-.542**	0.088
ptpn11								1	.351**	.301**	0.112	0.041
obrb									1	.446**	-.251*	.285*
socs3										1	-0.002	0.036
fkbp5											1	-.227*

Mc3r(TB/TB)

	CORT	acyl-ghrelin	leptin	*agrp*	*npy*	*pomc*	*cart*	*ptpn11*	*obrb*	*socs3*	*fkbp5*	*crh*
**CORT**	1	0.008	-0.018	.353**	.304**	-.230*	-0.103	0.214	-.348**	-.483**	.573**	-0.04
**acyl-ghrelin**		1	-.457**	0.158	0.184	-0.065	-.346**	-0.052	0.067	-0.154	-.255*	.229*
**leptin**			1	-.388**	-.482**	0.16	.370**	.307**	0.184	.302**	-0.048	-0.185
agrp				1	.916**	-0.093	-0.137	0.123	0.101	0.028	.245*	0.165
npy					1	-0.168	-.251*	-0.003	-0.101	-0.082	0.217	0.119
pomc						1	0.15	-0.159	.383**	.364**	-0.213	0.095
cart							1	0.083	0.074	.268*	0.009	0.035
ptpn11								1	0.213	0.132	0.208	-0.197
obrb									1	.496**	-.367**	.309**
socs3										1	-0.119	-0.167
fkbp5											1	-.343**
